# Identifying clinicopathological risk factors for regional lymph node metastasis in Chinese patients with T1 breast cancer: a population-based study

**DOI:** 10.3389/fonc.2023.1217869

**Published:** 2023-08-04

**Authors:** Gang Liu, Zeyu Xing, Changyuan Guo, Qichen Dai, Han Cheng, Xiang Wang, Yu Tang, Yipeng Wang

**Affiliations:** ^1^ Department of Breast Surgical Oncology, National Cancer Center/National Clinical Research Center for Cancer/Cancer Hospital, Chinese Academy of Medical Sciences and Peking Union Medical College, Beijing, China; ^2^ Department of Pathology, National Cancer Center/National Clinical Research Center for Cancer/Cancer Hospital, Chinese Academy of Medical Sciences and Peking Union Medical College, Beijing, China; ^3^ GCP center, National Cancer Center/National Clinical Research Center for Cancer/Cancer Hospital, Chinese Academy of Medical Sciences and Peking Union Medical College, Beijing, China

**Keywords:** breast cancer, small-sized tumor with extensive metastasis, hormone receptor, clinical pathology, lymph node metastasis

## Abstract

**Objectives:**

To analyze clinicopathological risk factors and regular pattern of regional lymph node metastasis (LNM) in Chinese patients with T1 breast cancer and the effect on overall survival (OS) and disease-free survival (DFS).

**Materials and methods:**

Between 1999 and 2020, breast cancer patients meeting inclusion criteria of unilateral, no distant metastatic site, and T1 invasive ductal carcinoma were reviewed. Clinical pathology characteristics were retrieved from medical records. Survival analysis was performed using Kaplan−Meier methods and an adjusted Cox proportional hazards model.

**Results:**

We enrolled 11,407 eligible patients as a discovery cohort to explore risk factors for LNM and 3484 patients with stage T1N0 as a survival analysis cohort to identify the effect of those risk factors on OS and DFS. Compared with patients with N- status, patients with N+ status had a younger age, larger tumor size, higher Ki67 level, higher grade, higher HR+ and HER2+ percentages, and higher luminal B and HER2-positive subtype percentages. Logistic regression indicated that age was a protective factor and tumor size/higher grade/HR+ and HER2+ risk factors for LNM. Compared with limited LNM (N1) patients, extensive LNM (N2/3) patients had larger tumor sizes, higher Ki67 levels, higher grades, higher HR- and HER2+ percentages, and lower luminal A subtype percentages. Logistic regression indicated that HR+ was a protective factor and tumor size/higher grade/HER2+ risk factors for extensive LNM. Kaplan−Meier analysis indicated that grade was a predictor of both OS and DFS; HR was a predictor of OS but not DFS. Multivariate survival analysis using the Cox regression model demonstrated age and Ki67 level to be predictors of OS and grade and HER2 status of DFS in stage T1N0 patients.

**Conclusion:**

In T1 breast cancer patients, there were several differences between N- and N+ patients, limited LNM and extensive LNM patients. Besides, HR+ plays a dual role in regional LNM. In patients without LNM, age and Ki67 level are predictors of OS, and grade and HER2 are predictors of DFS.

## 1 Introduction

Breast cancer is the malignant tumor with the highest incidence in the world and has one of the highest cancer mortality rates, accounting for 24.5% of all new malignant tumors in women (1). With the popularization of breast cancer screening, an increasing number of breast cancer cases are detected at an early stage (2). It is generally considered that increasing tumor size correlates with a high risk of lymph node involvement (3). However, some patients with small-sized breast cancer also have lymph node metastasis (LNM), even extensive LNM, when diagnosed (4). In general, patients with 4 or more metastatic lymph nodes (N2-3) are considered to have “extensive LNM” (5). It has been reported that approximately 27.8% of T1 breast cancer patients have LNM and approximately 0.7% extensive LNM at the time of diagnosis (4). Several studies have indicated that small tumors might be a surrogate for biologically aggressive disease, especially in extensive node-positive disease (4, 5). Therefore, it is of great significance to explore factors associated with LNM, the difference between limited LNM and extensive LNM, and effects on the survival of patients with small tumors.

Previous research has established that many factors are associated with LNM, including age at diagnosis, tumor size, hormone receptor (HR) and human epidermal growth factor receptor 2 (HER2) status, and Ki67 level (3, 6–8). Several studies have proven that race is also an important factor influencing LNM and survival (9–11). Nevertheless, the different risk factors between limited LNM and extensive LNM remain unclear. To date, there are only a few reports evaluating factors of LNM in the Chinese population, and those reports had limited sample sizes (12, 13).

In this study, we reviewed clinicopathological characteristics of T1 breast cancer patients in a Chinese population. We compared clinicopathological factors of LNM, including the difference between N- and N+, limited N+(N1) and extensive N+(N2/3). We also analyzed the effect on survival to gain a better understanding of LNM prediction and prognosis for patients with small tumors.

## 2 Materials and methods

### 2.1 Patients and materials

Breast cancer patients meeting the inclusion criteria of female, unilateral, no distant metastatic site, and T1 invasive ductal carcinoma in the Cancer Hospital of Chinese Academy of Medical Sciences were reviewed from January 1999 to September 2020. Patients with distant metastasis at diagnosis or primary malignant tumors of other organs in the past were excluded. Medical records were collected, including age and clinical pathology features, such as tumor size, grade, Ki67 level, HR status, HER2 status and lymph node involvement. In addition, we enrolled eligible patients from 2009 to 2017 as a survival analysis cohort to identify the effect of risk factors on the overall survival (OS) and disease-free survival (DFS) of T1 breast cancer patients. Survival time was calculated from the date of diagnosis to the occurrence of the event or the censoring date. The study was approved and a waiver of the informed consent of study participants was granted by the Ethics Committee of the Cancer Hospital of Chinese Academy of Medical Sciences.

### 2.2 Statistical analysis

Data were analyzed using SPSS software (version 23.0, SPSS Inc., Chicago, IL). Differences were evaluated using the χ^2^ test, the Fisher test, or the independent samples *t* test according to their characteristics. Multiple factor analysis was performed using logistic regression. To evaluate risk factors for LNM, we compared the characteristics of lymph node-negative (N-) patients with those of lymph node-positive (N+) patients. Then, we analyzed differences between N1 patients and N2/3 patients to validate the determination of limited LNM and extensive LNM. Survival analysis was carried out using Kaplan−Meier methods and an adjusted Cox proportional hazards model; p values less than 0.05 were considered statistically significant.

## 3 Results

### 3.1 Descriptive statistics

We enrolled 11,407 eligible breast cancer patients with stage T1 as a discovery cohort (**Table 1**): 7185 N- patients and 4222 N+ patients. Among the patients with N+ status, 2780 patients had N1 stage and 1442 patients N2/3.

**Table 1:** Basic characteristics of the discovery cohort with stage T1 patients.

Patient Characteristic	N- (n=7185)		N+ (n=4222)		Total (n=11407)	p
Age	52.05 ± 10.63		50.88 ± 10.66		51.62	<0.001
Tumor size	1.43 ± 0.45		1.57 ± 0.39		1.48	<0.001
Ki67	25.98% ± 19.71%		27.63% ± 19.84%		26.58%	<0.001
HR status						
positive	5594	62.6%	3348	37.4%	8942	0.041
negative	1460	64.9%	790	35.1%	2250	
HER2 status						
positive	1396	60.0%	932	40.0%	2328	<0.001
negative	5329	64.2%	2975	35.8%	8304	
Molecular Subtypes						
Luminal A ^a^	2301	67.2%	1124	32.8%	3425	<0.001
Luminal B ^b^	2728	59.6%	1846	40.4%	4574	
HER2-positive ^b^	562	60.1%	373	39.9%	935	
TNBC ^a^	849	68.3%	394	31.7%	1243	
Grade						
1^a^	888	76.1%	279	23.9%	1167	<0.001
2^b^	4399	62.5%	2662	37.7%	7061	
3^c^	1715	59.7%	1159	40.3%	2874	

HR, hormone receptor; HER2, human epidermal growth factor receptor 2; TNBC, triple negative breast cancer. ^a,b,c^ Each subscript letter denotes a subset of Molecular Subtypes categories whose row proportions do not differ significantly from each other at the 0.05 level.

### 3.2 Determination of LNM

Compared with N- patients, N+ patients were younger (50.88 ± 10.66 vs. 52.05 ± 10.63, p<0.001) (**Table 1**); the tumor size was larger in the N+ group (1.57 ± 0.39 vs. 1.43 ± 0.45, p<0.001), and Ki67 levels were higher (27.63% ± 19.84% vs. 25.98% ± 19.71%, p<0.001). We also compared differences in HR and HER2 status, molecular subtype and grade. The percentage of HR+ in the N+ group was higher than that in the HR- group (37.4% vs. 35.1%, p=0.041), and that of HER2+ in the N+ group was higher than that of HER2- (40.0% vs. 35.8%, p<0.001). In the N+ group, the percentage of luminal B and HER2-positive subtypes was higher (p<0.001) than that of luminal A and triple-negative breast cancer (TNBC) subtypes. There was also a greater percentage of higher grade in the N+ group (Grade 3/2/1: 40.3% vs. 37.7% vs. 23.9%, respectively, p<0.001). It is possible to hypothesize that these conditions are more likely to occur in patients with lymph node involvement, including younger age, larger tumor size, higher Ki67 level, HR+, HER2+, luminal B and HER2-positive subtypes, and higher grade.

Based on the difference between N- and N+ patients, logistic regression was conducted to identify risk factors for LNM, indicating that age, tumor size, grade, HR status, and HER2 status were significant indicators of lymph node involvement (**Table 2**). In contrast, age was a protective factor (OR, 0.987, 95%CI, 0.983 to 0.991, p<0.001). Tumor size was a risk factor (OR, 2.084, 95%CI, 1.862 to 2.332, p<0.001). Grade 2 (OR, 1.718, 95% CI, 1.451 to 2.035, p<0.001) and grade 3 (OR, 1.788, 95%CI, 1.452 to 2.201, p<0.001) were risk factors, as were HR positivity (OR, 1.249, 95%CI, 1.095 to 1.424, p=0.001) and HER2 positivity (OR, 1.168, 95%CI, 1.045 to 1.306, p=0.006).

**Table 2:** Logistic Regression on high risk factors of lymph node metastasis comparing to non-lymph node involvement in the discovery cohort with stage T1 patients.

	B	S.E.	Wald	p	OR	95% CI
Age	-0.013	0.002	35.475	**<0.001**	0.987	0.983-0.991
Tumor size	0.734	0.057	163.333	**<0.001**	2.084	1.862-2.332
Grade 1			40.030	<0.001	Ref	
Grade 2	0.541	0.086	39.325	**<0.001**	1.718	1.451-2.035
Grade 3	0.581	0.106	30.017	**<0.001**	1.788	1.452-2.201
Ki67	0.088	0.150	0.345	0.557	1.092	0.814-1.465
HR*	0.222	0.067	11.029	**0.001**	1.249	1.095-1.424
HER2*	0.156	0.057	7.482	**0.006**	1.168	1.045-1.306

HR, hormone receptor; HER2, human epidermal growth factor receptor 2; TNBC, triple negative breast cancer. CI, confidential interval. OR, Odd Ratio. The bold values mean statistically significantly.*, receptor status, positive vs negative.

### 3.3 Determination of extensive LNM compared with limited LNM

Compared with patients with limited LNM (1-3 lymph nodes involved, N1), those with extensive LNM (more than or equal to 4 lymph nodes involved, N2/3) had similar ages at diagnosis (50.75 ± 10.68 vs. 50.94 ± 10.65, p=0.593) (**Table 3**). Tumor size was larger in the extensive LNM group (1.60 ± 0.40 vs. 1.56 ± 0.39, p=0.001), and the Ki67 level was higher (28.84% ± 20.02% vs. 27.02% ± 19.73%, p=0.011). Then, we compared differences in HR/HER2 status, molecular subtypes and grade. In the extensive LNM group, the percentage of HR- was higher than that of HR+ (41.3% vs. 32.5%, p<0.001); in the extensive LNM group, the percentage of HER2+ was higher than that of HER2- (40.9% vs. 32.0%, p<0.001). The percentage of HER2-positive subtypes was also higher than that of luminal B and luminal A subtypes in the extensive LNM group (44.5% vs. 35.9% vs. 27.5%, p<0.001). The percentage of TNBC subtypes was higher than that of luminal A subtypes but was not different from that of luminal B and HER2-positive subtypes. There was also a higher percentage of higher grade in the extensive LNM group (grade 3/2/1: 40.3% vs. 32.3% vs. 21.1%, p<0.001). These results further support the hypothesis that these factors, including larger tumor size, higher Ki67 level, HR-, HER2+, non-luminal A subtypes and higher grade, increase risk of extensive LNM.

**Table 3:** Basic characteristics of the discovery cohort with stage T1N+ patients.

Patient Characteristic	limited LNM (n=2780)		extensive LNM (n=1442)		Total (n=4222)	p
Age	50.94 ± 10.65		50.75 ± 10.68		50.88	0.593
Tumor size	1.56 ± 0.39		1.60 ± 0.40		1.57	0.001
Ki67	27.02% ± 19.73%		28.84% ± 20.02%		27.63%	0.011
HR status						
positive	2260	67.5%	1088	32.5%	3348	<0.001
negative	464	58.7%	326	41.3%	790	
HER2 status						
positive	551	59.1%	381	40.9%	932	<0.001
negative	2022	68.0%	953	32.0%	2975	
Molecular Subtypes						
Luminal A ^a^	815	72.5%	309	27.5%	1124	<0.001
Luminal B ^b^	1184	64.1%	662	35.9%	1846	
HER2-positive ^c^	207	55.5%	166	44.5%	373	
TNBC ^b,c^	248	62.9%	146	37.1%	394	
Grade						
1 ^a^	220	78.9%	59	21.1%	279	<0.001
2 ^b^	1803	67.7%	859	32.3%	2662	
3 ^c^	692	59.7%	467	40.3%	1159	

HR, hormone receptor; HER2, human epidermal growth factor receptor 2; TNBC, triple negative breast cancer. ^a,b,c^ Each subscript letter denotes a subset of Molecular Subtypes categories whose row proportions do not differ significantly from each other at the 0.05 level.

Based on the difference between limited LNM and extensive LNM patients, logistic regression was conducted to identify risk factors for extensive LNM. Logistic regression indicated that tumor size, grade, HR status, and HER2 status were significant indicators of extensive LNM (**Table 4**). Tumor size was a risk factor (OR, 1.240, 95%CI, 1.019 to 1.510, p=0.032). Grade 2 (OR, 1.731, 95%CI, 1.216 to 2.463, p=0.002) and grade 3 (OR, 2.225, 95%CI, 1.494 to 3.312, p<0.001) were also risk factors compared to grade 1. HR positivity was a protective factor (OR, 0.016, 95%CI, 0.620 to 0.952, p=0.016) and HER2 positivity a risk factor for extensive LNM (OR, 1.212, 95%CI, 1.012 to 1.451, p=0.036).

**Table 4:** Logistic Regression on high risk factors of extensive lymph node metastasis comparing with limited lymph node metastasis in the discovery cohort with stage T1N+ patients.

	B	S.E.	Wald	p	OR	95% CI
Age	0.000	0.004	0.013	0.908	1.000	0.993-1.007
Tumor size	0.215	0.100	4.618	**0.032**	1.240	1.019-1.510
Grade 1			15.975	<0.001	Ref	
Grade 2	0.549	0.180	9.295	**0.002**	1.731	1.216-2.463
Grade 3	0.800	0.203	15.500	**<0.001**	2.225	1.494-3.312
Ki67	-0.253	0.238	1.133	0.287	0.776	0.487-1.237
HR*	-0.263	0.109	5.798	**0.016**	0.768	0.620-0.952
HER2*	0.192	0.092	4.379	**0.036**	1.212	1.012-1.451

HR, hormone receptor; HER2, human epidermal growth factor receptor 2; TNBC, triple negative breast cancer. CI, confidential interval. OR, Odd Ratio. The bold values mean statistically significantly.*, receptor status, positive vs negative.

### 3.4 Effects of different clinical factors on survival

There were 3484 patients in the survival analysis cohort (**Table S1**). Age at diagnosis was 52.23 ± 10.46 years. The mean tumor size was 1.45 ± 0.46 cm, and the Ki67 level was 27.35% ± 20.50%. The percentage of HR+ patients was 76.1%, and that of HER2+ patients was 21.1%. There were approximately 31.5% luminal A, 40.1% luminal B, 8% HER2-positive and 12.1% TNBC subgroup patients. Percentages of grade 1, grade 2 and grade 3 were 12.1%, 60.6%, and 25.3%, respectively.

**Figure 1:**
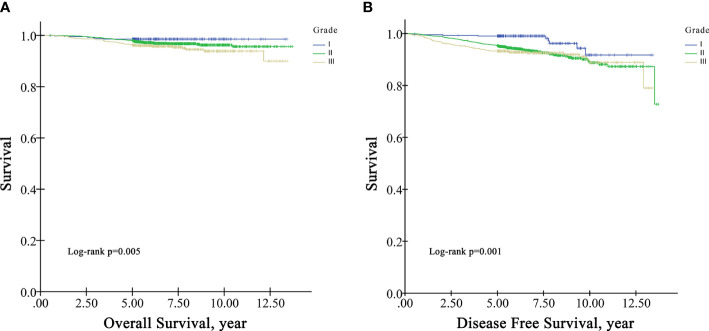
Univariate Kaplan−Meier survival plots for grade. **(A)** for OS; **(B)** for DFS.

**Figure 2:**
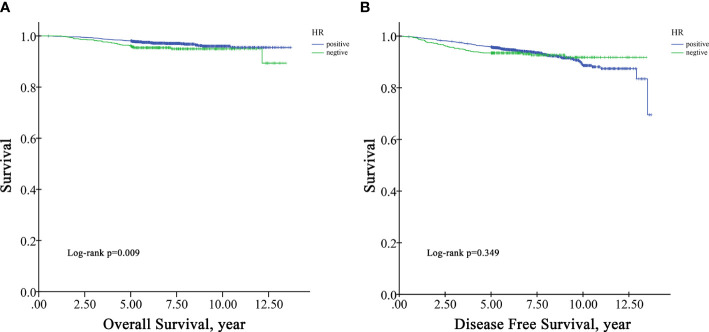
Univariate Kaplan−Meier survival plots for HR. **(A)** for OS; **(B)** for DFS.

**Figure 3:**
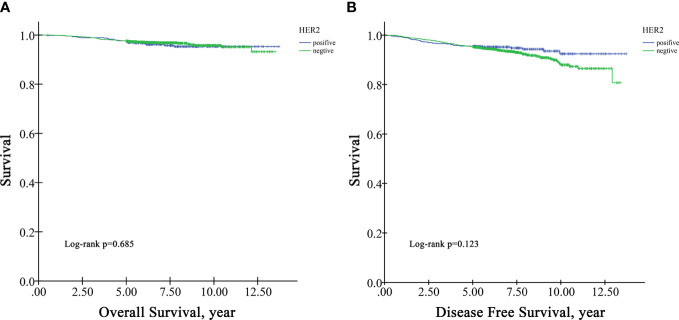
Univariate Kaplan−Meier survival plots for HER2. **(A)** for OS; **(B)** for DFS.

In the survival analysis cohort, the mean follow-up time after diagnosis was 6.60 years, and the median follow-up time was 5.71 years. There were 107 deaths and 216 patients with disease progression in this cohort. The OS rate at five years was 97.5%, and the DFS rate at five years was 95.13%. According to Kaplan−Meier survival analysis, higher grade patients had shorter OS (log-rank p=0.005) and DFS (log-rank p=0.001) than lower grade patients (**Figure 1**). Moreover, patients with negative HR had shorter OS than those with positive HR (log-rank p=0.009) (**Figure 2A**), but DFS was not significantly different between these two groups (log-rank p=0.349) (**Figure 2B**), and there was no significant difference in OS and DFS between the HER2-positive group and the HER2-negative group (**Figure 3**).

According to multivariate survival analysis using the Cox regression model, several factors were independent predictors of OS and DFS in the validation cohort (**Table 5**). Age at diagnosis (HR=1.022, 95%CI=1.001-1.043, p=0.045) and Ki67 level (HR=6.367, 95%CI=1.863-21.761, p=0.003) were predictors of OS and higher grade (Grade II, HR=3.715, 95%CI=1.500-9.199, p=0.005. grade III, HR=4.321, 95%CI=1.612-11.581, p=0.004) and HER2 status (HR=0.607, 95%CI=0.402-0.917, p=0.018) were predictors of DFS in stage T1N0 patients.

**Table 5:** The effects of different clinical factors on Overall Survival and Disease Free Survival in the survival analysis cohort with stage T1N0 patients.

	OS						DFS					
	B	SE	Wald	p	hazard ratio	95.0% CI	B	SE	Wald	p	hazard ratio	95.0% CI
AGE	0.021	0.011	4.028	**0.045**	1.022	1.001-1.043	-0.009	0.008	1.420	0.233	0.991	0.976-1.006
Tumor size	0.021	0.272	0.006	0.939	1.021	0.600-1.738	0.387	0.202	3.674	0.055	1.472	0.991-2.187
Grade 1			1.406	0.495	Ref				8.687	0.013	Ref	
Grade 2	0.610	0.532	1.311	0.252	1.840	0.648-5.225	1.312	0.463	8.046	**0.005**	3.715	1.500-9.199
Grade 3	0.687	0.599	1.317	0.251	1.988	0.615-6.425	1.464	0.503	8.467	**0.004**	4.321	1.612-11.581
Ki67	1.851	0.627	8.715	**0.003**	6.367	1.863-21.761	0.444	0.469	0.896	0.344	1.559	0.621-3.912
HR*	0.157	0.293	0.286	0.593	1.170	0.658-2.080	0.161	0.219	0.540	0.463	1.174	0.765-1.803
HER2*	-0.172	0.271	0.401	0.527	0.842	0.495-1.433	-0.499	0.210	5.639	**0.018**	0.607	0.402-0.917

HR, hormone receptor; HER2, human epidermal growth factor receptor 2; TNBC, triple negative breast cancer. CI, confidential interval. The bold values mean statistically significantly.*, receptor status, positive vs negative.

## 4 Discussion

Small-sized breast cancer with LNM is a special kind of aggressive breast cancer, especially with extensive LNM. In this study, we sought to determine risk factors and the regular pattern of LNM in small-sized tumors (defined as T1 tumors), between no metastasis (N-) to metastasis (N+), and between limited metastasis (N1) to extensive metastasis (N2/N3). We also sought to determine whether these risk factors affect the OS and DFS of patients with small tumors without LNM.

According to our analysis, younger age at diagnosis is a risk factor for lymph node involvement. However, age was not found to be a risk factor for extensive LNM compared to limited LNM. There were similarities between the findings of this study and those by other researchers (7, 14–16). Younger age has also been reported as a risk factor for distant metastasis (17). Our study found age to be an independent risk predictor of OS in patients with small tumors without LNM. These results are in line with those of previous studies (5, 18, 19).

In our study, tumor size was associated with LNM, including cases without LNM to tumors with extensive LNM. Some studies have postulated a convergence between tumor size and LNM (15, 20). T1N0 breast cancer has good prognosis, and our study found that tumor size was not a predictor of OS or DFS in these patients. Nonetheless, for tumors with LNM, small tumor size is associated with survival (5).

Our study results also showed Ki67 level to be a risk factor for LNM. The importance of Ki67 levels in predicting LNM in patients with small tumors has been noted (8, 21, 22). One unanticipated finding was that the Ki67 level was an independent predictor of OS in patients with small tumors without LNM. Previous studies have indicated that the level of Ki67 is an independent prognostic factor of breast cancer-specific survival (BCSS) and DFS in breast cancer patients (23, 24), and our study confirmed the value of the level of Ki67 in prediction of survival in small-sized tumor patients.

Importantly, high histological grade was associated with LNM in our study. Similar to the role of tumor size, higher grade increased LNM risk from limited metastasis to extensive involvement. In addition, grade was a predictor of OS and DFS in patients with small tumors without LNM. In accordance with the present results, previous studies have demonstrated that high grade indicates a high labeling index, rapid replication, and a greater early relapse rate than low grade (22, 25). Therefore, high grade can be used for predicting LNM and survival in patients with small tumors.

The most striking and unanticipated finding was that HR+ plays a dual role in regional LNM: although HR+ status was a risk factor for LNM, in N+ patients, HR+ status was a protective factor against extensive LNM. Consistent with the literature, there are some studies indicating that HR+ status is a protective factor against LNM (7, 22), but there are also several studies showing that HR+ status is a risk factor, as we observed in our study (6, 26, 27). To our knowledge, this is the first study to identify this dual role in regional LNM. It is possible to hypothesize that HR+ status is likely to increase but also restrict risk of LNM. One possible explanation is that estrogen is important for tumor growth, and required for intratumoral lymphangiogenesis which could increase the risk of LNM, but in the lymph node microenvironment, ER is dysfunctional and the sensitivity is altered, which could also influence LNM (28). We also found that HR+ status was a protective predictor of OS in univariate survival analysis but failed to find a tendency in multivariate survival analysis. In accordance with the present results, previous studies have indicated that HR positivity is a protective factor for DFS and OS in small-tumor patients (18, 29). Further work including subgroup analysis is needed to confirm these findings.

In our study, we found HER2+ status to be a risk factor for LNM with small tumors. These results mirror those of previous studies (8, 26). We also found HER2+ status to be an independent protective factor of DFS in multivariate survival analysis. HER2+ status is reportedly associated with poor prognosis (30, 31), especially in patients with stage T3/T4 tumors (31). For small-sized tumors, Gonzalez-Angulo et al. (2009) concluded that HER2 positivity is an independent risk factor for DFS in T1a and T1b breast cancer without node positivity (29). This discrepancy might be attributed to racial differences. Indeed, several studies have indicated that ethnicity is associated with survival of breast cancer patients (11, 18). We also observed higher DFS in the HER2+ group in another hospital in China (32). Another possible alternative explanation of our findings is that anti-HER2 therapy increases the DFS of patients with small tumors. In general, patients with HER2 positivity and stage T1b/T1c should undergo anti-HER2 therapy. Several studies have indicated that HER2+ patients have increased DFS after anti-HER2 therapy (33), but few prospective studies have compared the prognosis of HER2- patients and HER2+ patients treated with anti-HER2 therapy. Further research is required to evaluate the impact of HER2+ status.

There are several limitations in our study. First, information for real-world treatment was not reviewed when we conducted survival analysis. Although we only included patients with small-sized tumors without LNM, not all of the patients underwent standard treatment, which may lead to bias in survival analysis. Second, the sample size used for survival analysis was small, possibly influencing its significance, especially for the HER2+ group. Larger samples are needed for replication.

## 5 Conclusion

Our study describes the first and largest cohort of small-sized breast cancer patients in a single center in China. We reveal several factors associated with LNM for T1 tumors, including age, tumor size, Ki67 level, grade, and HR and HER2 status. We found that HR+ status plays a dual role in regional LNM. We also evaluated the effect of the above risk factors on the survival of T1 tumor patients. Univariate analysis indicated grade and HR status to be predictors of OS, with grade also being a predictor of DFS. Multiple regression analysis showed that age and Ki67 level are predictors of OS and that grade and HER2 are predictors of DFS in patients with small-sized tumors without LNM. The findings of our study have a number of important implications for prediction of LNM and survival in patients with small tumors.

## Data availability statement

The raw data supporting the conclusions of this article will be made available by the authors, without undue reservation.

## Ethics statement

The study was approved and a waiver of the informed consent of study participants was granted by the Ethics Committee of the Cancer Hospital of Chinese Academy of Medical Sciences.

## Author contributions

GL and YW contributed to the conception, supervision, and funding of the study. GL, CG and ZX collected the medical data. QD and HC conducted the data analyses. GL, CG and ZX wrote the original draft. XW and YT were responsible for the critical revision of the manuscript or important intellectual content. All authors contributed to the article and approved the submitted version.
