# Supplementary material for: Identifying clinicopathological risk factors for regional lymph node metastasis in Chinese patients with T1 breast cancer: a population-based study
Source: Front Oncol. 2023 Aug 4;13:1217869. doi: 10.3389/fonc.2023.1217869 (PMC10436470; doi:10.3389/fonc.2023.1217869)
Supplement: Supplementary file 1 [file Table_1.docx]

Table S1. Basic characteristics of the survival analysis cohort with stage T1N0 patients.

| Patient  Characteristic | N0  (n=3484) | |
| --- | --- | --- |
| Age | 52.23±10.46 | |
| Tumor size | 1.45±0.46 | |
| Ki67 | 27.35%±20.50% | |
| HR status |  | |
| positive | 2653 | 76.1% |
| negative | 723 | 20.8% |
| missing | 108 | 3.1% |
| HER2 status |  | |
| positive | 734 | 21.1% |
| negative | 2443 | 70.1% |
| missing | 307 | 8.8% |
| Molecular Subtypes |  | |
| Luminal A | 1097 | 31.5% |
| Luminal B | 1398 | 40.1% |
| HER2-positive | 277 | 8% |
| TNBC | 422 | 12.1% |
| missing | 290 | 8.3% |
| Grade |  | |
| 1 | 422 | 12.1% |
| 2 | 2111 | 60.6% |
| 3 | 880 | 25.3% |
| missing | 71 | 2.0% |

HR, hormone receptor. HER2, human epidermal growth factor receptor 2. TNBC, triple negative breast cancer.
